# Assessment of health state utilities associated with adult and pediatric acid sphingomyelinase deficiency (ASMD)

**DOI:** 10.1007/s10198-023-01667-7

**Published:** 2024-02-27

**Authors:** Louis S. Matza, Katie D. Stewart, Marie Fournier, Donna Rowen, Robin Lachmann, Maurizio Scarpa, Eugen Mengel, Travis Obermeyer, Evren Ayik, Fernando Laredo, Ruth Pulikottil-Jacob

**Affiliations:** 1 Patient-Centered Research, Evidera, 7101 Wisconsin Avenue, Suite 1400, Bethesda, MD 20814 USA; 2 Sanofi, Chilly-Mazarin, France; 3 School of Health and Related Research, University of Sheffield, Sheffield, UK; 4 University College London Hospitals, London, UK; 5 Centro Coordinamento Regionale Malattie Rare, Azienda Sanitaria Universitaria del Friuli Centrale, Udine, Italy; 6 SphinCS-Institute of Clinical Science for Lysosomal Storage Diseases, Hochheim, Germany; 7 NNPDF, Charlotte, NC USA; 8 Fresno, CA USA; 9 Sanofi, São Paulo, Brazil; 10 Sanofi, Thames Valley Park, Reading, UK

**Keywords:** Utility, Acid sphingomyelinase deficiency, Rare disease, Time trade-off, TTO, Pediatric utility, I. Health, Education, and Welfare, I00 General, I1 Health, I10 General

## Abstract

**Introduction:**

Acid sphingomyelinase deficiency (ASMD) type B is a rare genetic disorder leading to enlargement of the spleen and liver, pulmonary dysfunction, and other symptoms. Cost-utility analyses are often conducted to quantify the value of new treatments, and these analyses require health state utilities. Therefore, the purpose of this study was to estimate utilities associated with varying levels of severity of adult and pediatric ASMD type B.

**Methods:**

Seven adult and seven child health state vignettes describing ASMD were developed based on published literature, clinical trial results, and interviews with clinicians, patients with ASMD, and parents of children with ASMD. The health states were valued in time trade-off interviews with adult general population respondents in the UK.

**Results:**

Interviews were completed with 202 participants (50.0% female; mean age = 41.3 years). The health state representing ASMD without impairment had the highest mean utility for both the adult and child health states (0.92/0.94), and severe ASMD had the lowest mean utility (0.33/0.45). Every child health state had a significantly greater utility than the corresponding adult health state. Differences between adult/child paired states ranged from 0.02 to 0.13. Subgroup analyses explored the impact of parenting status on valuation of child health states.

**Discussion:**

Greater severity of ASMD was associated with lower mean utility. Results have implications for valuation of pediatric health states. The resulting utilities may be useful in cost-utility modeling estimating the value of treatment for ASMD.

**Supplementary Information:**

The online version contains supplementary material available at 10.1007/s10198-023-01667-7.

## Introduction

Acid sphingomyelinase deficiency (ASMD; historically known as Niemann–Pick disease types A, A/B, and B) is a rare genetic disorder that results in an accumulation of sphingomyelin. Whereas ASMD type A is usually diagnosed during infancy, with rapid progression to death during early childhood [1], ASMD type B has a later onset and a slower rate of progression [2–6]. Commonly reported signs and symptoms of ASMD type B include enlargement of the spleen and liver, pulmonary dysfunction, dyspnea, dyslipidemia, fatigue, and pain [3, 6–8]. ASMD type B is also associated with limited physical activity [9], growth deficits in children, and declines in physical function, such as walking and standing [6, 10], as well as impact on social relationships [9], psychological functioning [9], and general health-related quality of life [11].

Olipudase alfa, an enzyme replacement therapy, is the first disease-modifying therapy for non-central nervous system manifestations of ASMD in children and adults [12]. Olipudase alfa treatment is associated with improvements in organ volumes, pulmonary function, hematologic parameters, dyslipidemia, and in children, catch-up growth [13–16]. As with any new treatment, cost-effectiveness analyses may be useful in a range of countries to examine the value of olipudase alfa and inform decision-making about healthcare resource allocation [17–19]. Submissions to health technology assessment (HTA) agencies typically include a type of cost-effectiveness model called a cost-utility analysis (CUA). In a CUA, health-related quality of life is quantified in terms of health state utilities, which are values on a scale anchored to 1 (full health) and 0 (dead) representing the strength of preference for health states. HTA agencies often prefer that CUAs incorporate utilities derived from generic preference-based instruments such as the EQ-5D [20–22].

For rare diseases like ASMD, however, generic instruments completed by patients may not be sensitive or feasible, and alternate approaches may be necessary [23, 24]. To derive utilities from patient-completed instruments such as the EQ-5D, a sufficiently large sample of patients is needed to represent the health states that will be included in economic models. With a rare disease such as ASMD, it may not be feasible to recruit a large enough sample of patients to obtain a reliable utility estimate for every health state needed in a CUA [25, 26]. This is further complicated by onset of ASMD in childhood because there are challenges associated with obtaining health-related quality of life data in children that are consistent with adult data [27, 28]. In addition, generic instruments may not assess concepts that are important to patients with specific rare diseases like ASMD [25]. For example, available generic instruments lack key aspects of ASMD, such as deterioration in pulmonary function and symptoms related to spleen volume, including bleeding and bruising [6, 8, 11, 29].

When generic preference-based instruments are not appropriate, feasible, or sufficient, some HTA agencies such as the National Institute for Health and Care Excellence allow for the use of alternative methods to estimate utilities [21]. With rare diseases, vignette-based methodology is often used to estimate utilities for use in HTA submissions [24, 30–37]. In these studies, health state vignettes can be drafted based on best available evidence and valued in preference-based tasks by general population respondents [23, 37].

The purpose of the current vignette-based study was to estimate utilities associated with varying severity of ASMD type B (subsequently referred to as ASMD). Because this condition typically presents in childhood and persists into adulthood [2, 8, 11, 38], CUAs of treatments for ASMD will require utilities associated with both adult and pediatric health states. Therefore, this study was designed to estimate utilities for two parallel sets of health states, one set of health states describing adults with ASMD and another describing children. These data provide a unique opportunity to examine utility differences between health state vignettes representing adults and children with the same disease.

## Methods

### Overview of study design

To determine whether a vignette-based study would be necessary for estimating utilities associated with ASMD, analyses were conducted using data from two generic preference-based instruments (EQ-5D-5L, SF-6D) administered in a clinical trial for treatment of ASMD [13, 15]. At baseline, utilities derived with these two generic instruments did not correspond to ASMD severity as assessed by measurements of organ volume and pulmonary function, suggesting that these generic instruments were not sensitive to severity of illness or impact on quality of life in patients with ASMD. Furthermore, EQ-5D-5L and SF-6D data were limited by the relatively small sample size of a clinical trial conducted in patients with a rare disease.

Due to these limitations, an alternative utility assessment approach was necessary [23]. The vignette-based method was selected for this study because it is well-suited for estimating utilities associated with the specific symptoms and impairment observed in patients with rare diseases [31–36]. This approach also overcomes the sample size limitations associated with rare diseases because vignettes can be valued by general population respondents, rather than people with the relevant disease.

Following recently published recommendations on vignette development and valuation [23, 37], 14 health state vignettes were developed to represent ASMD based on best available evidence, including input from clinicians, patients, and caregivers. Utilities for these health states were elicited in a time trade-off (TTO) task with a 10-year time horizon. All interviews were conducted in person by trained interviewers with adult general population respondents in March 2022 in London, England. All participants provided informed consent before completing the study procedures. All study procedures and materials were approved by an independent institutional review board (Ethical and Independent Review Services; Study Number 21178-01).

### Health state development

Health state vignettes described varying levels of ASMD severity for adults and children. These health states were developed based on a review of clinical trial data, consultation with clinicians specializing in ASMD, interviews with adults with ASMD, and interviews with caregivers of children with ASMD. A targeted literature search was performed to inform the development of the clinician, patient, and caregiver interview guides and to identify symptoms and areas of impact associated with ASMD to be included in the health states [2–11, 39–42].

To inform content of the health states, multiple rounds of interviews were conducted with three clinicians from England, Germany, and Italy, including two physicians specializing in metabolic diseases (one in adults and one in pediatrics) and a physician specializing in lysosomal storage disorders in both adults and children. Each clinician had more than 20 years of clinical experience in managing patients with ASMD. In the initial interviews, the advisors were asked to describe typical patient experiences with ASMD. These descriptions were used to develop the first draft of the health states, which were then reviewed and refined in subsequent interviews to ensure the descriptions were clear and accurate representations of the typical patient experience.

Interviews were also conducted with six adults with ASMD and two caregivers of children with ASMD. These patients and caregivers provided feedback on the health state descriptions. Each respondent was asked to review and comment on the health state most closely matching their own current condition (or their child’s current condition), as well as any health states corresponding to their previous experiences with ASMD. Health states were revised based on their input, and these revisions were discussed with and approved by the clinicians.

The final set of 14 health states included seven adult and seven pediatric vignettes describing typical experiences with ASMD at varying levels of severity. Although ASMD is associated with a variety of symptoms [8, 11], the health states focused on pulmonary dysfunction, enlargement of the spleen and liver, and the impact of these impairments on quality of life. Pulmonary dysfunction and spleen/liver enlargement were prioritized because they are key manifestations of ASMD that are associated with notable impact on patients’ lives [6, 8, 11, 29, 39–41].

The seven health states in the adult and pediatric sets included combinations of pulmonary dysfunction severity and spleen/liver enlargement (summarized in Table 1). These combinations of symptom severity were selected based on discussions with the clinicians and review of clinical trial data to identify the combinations of symptoms commonly experienced by patients. For example, there was no health state representing severe pulmonary dysfunction without spleen/liver enlargement because the clinicians reported that they do not see this combination in their patients. This clinical opinion was supported by baseline data from two clinical trials [13, 15] and data from a natural history study of ASMD [43]. Among the 84 patients with ASMD across these three studies, none had severe pulmonary dysfunction without spleen/liver enlargement, and only 2.4% had severe spleen/liver enlargement without pulmonary dysfunction.

**Table 1:** List of health states

Adult health states	Child health states	Description	Pulmonary function	Spleen and liver size
A1	C1	ASMD without symptoms or impairment	**No impairment** DLco ≥ 80%	**Normal** Spleen volume < 6 MN Liver volume normal
A2	C2	ASMD with mild-to-moderate pulmonary dysfunction	**Mild-to-moderate impairment** DLco ≥ 40% to 79%	**Normal** Spleen volume < 6 MN Liver volume normal
A3	C3	ASMD with moderate spleen/liver enlargement	**No impairment** DLco ≥ 80%	**Moderately enlarged** Spleen volume ≥ 6 to 15 MN Moderate liver enlargement
A4	C4	Mild-to-moderate ASMD (combination of moderate impairment described in A2/C2 and A3/C3)	**Mild-to-moderate impairment** DLco ≥ 40% to 79%	**Moderately enlarged** Spleen volume ≥ 6 to 15 MN Moderate liver enlargement
A5	C5	ASMD with mild-to-moderate pulmonary dysfunction with severe spleen/liver enlargement	**Mild-to-moderate impairment** DLco ≥ 40% to 79%	**Very enlarged** Spleen volume > 15 MN Severe liver enlargement
A6	C6	ASMD with severe pulmonary dysfunction with moderate spleen/liver enlargement	**Severe impairment** DLco < 40%	**Moderately enlarged** Spleen volume ≥ 6 to 15 MN Moderate liver enlargement
A7	C7	Severe ASMD (combination of severe impairment described in A5/C5 and A6/C6)	**Severe impairment** DLco < 40%	**Very enlarged** Spleen volume > 15 MN Severe liver enlargement

*ASMD* acid sphingomyelinase deficiency, *DLco* diffusing lung capacity, *MN* multiples of normal

The health states were presented to respondents on individual cards with bullet point descriptions organized into sections with headings for “Breathing,” “Spleen and Liver,” and “Quality of Life.” The “Quality of Life” section included subheadings for “activities,” “infections and hospitalizations,” “appearance,” and “emotional impact.” The final health states are presented in the Supplementary Material.

### Participants

This study was conducted with a sample of general population respondents, consistent with HTA preferences for the general population perspective in utility elicitation [21]. Participants were recruited using digital social media marketing (e.g., via Facebook, Twitter, Google). Potential participants who responded with interest were screened by phone for eligibility. All participants were required to be over 18 years old, a resident of the UK, able to understand the assessments as judged by the investigator, able and willing to give electronic consent, and able to complete the protocol requirements. Efforts were made to recruit a sample of respondents reflective of the UK general population with regard to gender, age, racial/ethnic background, and rate of unemployment. For the safety of interviewers and participants, all study personnel and participants were required to be vaccinated against COVID-19 and masked during the interview.

### Pilot study

A pilot study was conducted with 25 general population participants in London, England. The purpose of this pilot study was to evaluate the health states and the utility assessment procedures to ensure they were clear to respondents. Participants were asked to complete the utility valuation and provide feedback. Health states and procedures were revised based on this feedback to improve clarity and ease of understanding in the larger valuation study. The results from pilot interviews were not included in the subsequent analysis.

### Utility interview procedures and scoring

The health states and procedures finalized in the pilot study were used to elicit utilities in the main study. Interviewers trained in TTO methods conducted one-on-one interviews following a semi-structured interview guide using TTO booklets and health state cards. Participants were randomized to begin with either the adult or pediatric health states first, followed by the other set.

Participants were first introduced to the health states and shown a background description of either adult or pediatric ASMD, according to their health state order assignment. Then, the first set of seven health states (i.e., either adult or child) was presented in random order, and participants were asked to rank the seven health states in order of preference (i.e., from most preferable to least preferable). Health states were labeled with letters to facilitate discussion during the interviews, but the order of the letters did not correspond to the severity of the health states. After ranking the health states and explaining their preferences, participants valued the health states in the TTO task. Then, participants completed the ranking and TTO tasks for the second set of health states.

To elicit utilities for the adult health states, participants valued the health states in a TTO task with a 10-year time horizon following procedures described extensively in previous publications [17, 44]. The specified amount of time in the health state (i.e., the time horizon) varies across TTO utility elicitation studies, and no specific time horizon is universally preferred or considered to be more “correct” [44–47]. The most commonly used TTO time horizon appears to be 10 years, which was used in the influential Measurement and Valuation of Health study that identified utilities of EQ-5D health states [48, 49], the EQ-VT protocol for valuation of EQ-5D-5L states, the international valuation protocol for valuing pediatric health states derived from the EQ-5D-Y [50], and a wide range of vignette-based studies [47, 51–56]. To maximize comparability with previous research, the 10-year time horizon was selected for the current study.

For each health state, participants were offered a series of choices between a 10-year period in the health state being rated and varying amounts of time in full health. For the adult health states, the TTO task was presented in the context of the respondent’s own life (“Which life would you prefer? You can choose Life 1, Life 2, or they can be equal.”). Choices were presented in 6-month (5%) increments, alternating between longer and shorter periods of time in full health. The increment of 6 months was selected to allow for a reasonable level of sensitivity to differences between the health states, without assuming a potentially unrealistic degree of precision that would be associated with smaller increments.

There is no consensus regarding the best approach for eliciting utilities for pediatric health states [28, 57, 58]. Studies vary with regard to the type of sample valuing the health states (e.g., parents, general population adults, or children) as well as the framing of the utility valuation task (e.g., valuing health states for one’s own child, yourself as a child, or a hypothetical unknown child). The current study used an approach similar to a recent study involving EQ-5D-Y health states [50]. General population adult respondents were asked to imagine a child of a specific age, but the child’s identity was not specified. The age of 8 years old was selected for the current study because it was the mean age of pediatric participants at baseline in the ASCEND-Peds trial of treatment for ASMD [13]. The TTO task was framed as follows: “Imagine an 8-year-old child with a rare genetic disorder. For this child, which life do you think would be preferable? Life 1, Life 2, or are Life 1 and Life 2 about equal?” Other than this re-framing, the TTO procedures for pediatric health states were the same as the procedures described above for the adult health states. After completing the TTO task, participants were asked if they were thinking about a specific child during the TTO.

Each health state perceived to be better than “dead” received a utility value (*u*) on a scale with anchors of dead (0) and full health (1) based on the point of indifference between *y* years in the health state being valued and *x* years in full health (followed by dead). Utility was calculated as *u* = *x*/*y*. When participants preferred “dead” over a health state, the task and scoring procedures were altered as described in previous literature [59]. Participants were offered a choice between dead (choice 1) and a 10-year life span (choice 2) beginning with varying amounts of time in the health state being rated, followed by full health for the remainder of the life span. The resulting negative utility scores were calculated with the bounded scoring approach (*u* = *−* *x*/10, where *x* is the number of years in full health).

### Statistical analysis procedures

Statistical analyses were completed with SAS (version 9.4, SAS Institute, Cary, NC, USA). Descriptive statistics were used to summarize demographic data, health state rankings, and utilities. Categorical variables are summarized as frequencies and percentages, while means and standard deviations are reported for continuous variables. Paired *t* tests were conducted to test whether there were statistically significant pairwise differences between heath state utilities (e.g., the utility of the health state representing ASMD without impairment vs. severe ASMD). Independent *t* tests were conducted to test for differences in utility by age, gender, and parenting status.

**Table 2:** Demographic and clinical characteristics

Characteristics	Descriptive statistics (*n* = 202)
Age, mean years (SD)	41.3 (14.4)
Gender, *n* (%)	
Male	99 (49.0%)
Female	101 (50.0%)
Nonbinary	2 (1.0%)
Ethnic/racial background, *n* (%)	
Asian/Asian British	20 (9.9%)
Black/African/Caribbean/Black British	5 (2.5%)
White	167 (82.7%)
Mixed/multiple ethnic groups	7 (3.5%)
Other	3 (1.5%)
Marital status, *n* (%)	
Single	114 (56.4%)
Married/cohabitating/living with a partner	61 (30.2%)
Other^a^	27 (13.4%)
Do you have any children?	
No, *n* (%)	151 (74.8%)
Yes, *n* (%)	51 (25.2%)
If yes, how many? Mean (SD)	1.7 (0.8)
If yes, how many of these children still depend on you to care for them? Mean (SD)	0.8 (0.8)
Employment status, *n* (%)	
Full-time work	92 (45.5%)
Part-time work	40 (19.8%)
Other^b^	70 (34.7%)
Education level, *n* (%)	
University degree	146 (72.3%)
No university degree	56 (27.7%)

*SD* standard deviation

^a^Other marital status includes divorced (*n* = 19), separated (*n* = 4), widowed (*n* = 1), and other (not specified) (*n* = 3)

^b^Other employment status includes student (*n* = 20), unemployed (*n* = 19), retired (*n* = 14), homemaker (*n* = 7), and other (not specified) (*n* = 10)

## Results

### Sample characteristics

A total of 221 participants were scheduled, and 203 attended their interviews. One of the 203 had difficulty understanding the utility procedures and was unable to provide valid data. Therefore, the analyses were conducted with a sample of 202 participants. The sample reported gender as 50.0% female, 49.0% male, and 1.0% nonbinary, with a mean age of 41.3 years (Table 2). About one-quarter of the sample reported having children (25.2%). The most commonly reported health conditions were anxiety (33.2%), depression (27.2%), asthma (10.9%), and arthritis (8.9%). No participants reported having ASMD, and one participant (0.5%) reported knowing someone who has been diagnosed with ASMD.

### Health state rankings and preferences

In the introductory task, participants ranked the seven adult health states (A1 to A7) and the seven child health states (C1 to C7) separately in order of preference. Rankings, which ranged from 1 (most preferable health state) to 7 (least preferable health state), followed the same pattern for the adult and child health states. The health states describing ASMD without impairment (A1, C1) were most preferred for all participants. The health states describing severe ASMD were least preferred by nearly all participants (99.5% for A7; 100% for C7).

Preferences for the intermediate health states also followed a logical order. The health states with only one symptom at mild-to-moderate severity (A2, A3, C2, C3) were rated as second and third in preference by nearly all participants (99.5%), although the order varied between these pairs of health states (i.e., A2 vs. A3 and C2 vs. C3). Mild-to-moderate ASMD was ranked fourth by nearly all participants (99.5% for both A4 and C4). The health states with one mild-to-moderate symptom and one severe symptom (A5, A6, C5, C6) were ranked as fifth and sixth by nearly all participants (99.5%), with varying order between these pairs of health states (i.e., A5 vs. A6 and C5 vs. C6).

### Health state utilities

**Fig. 1:**
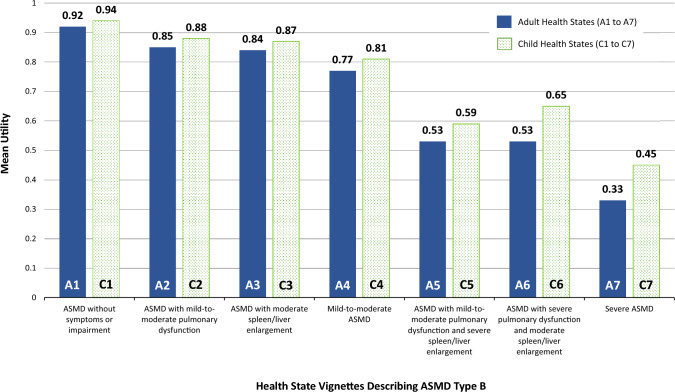
Health state utilities

Health state utilities are presented in Table 3 and Fig. 1. Mean utilities followed a logical order, with utility decreasing as symptom severity increased. ASMD without symptoms or impairment had the highest utility for both the adult (A1) and child health states (C1) (0.92 and 0.94, respectively). Severe ASMD had the lowest utility for both the adult (A7) and child health states (C7) (0.33 and 0.45, respectively). Every child health state had a significantly greater utility than the corresponding adult health state. Differences between adult/child pairs ranged from 0.02 (A1 vs. C1) to 0.13 (A6 vs. C6), and *P* values for these seven comparisons ranged from 0.04 to < 0.0001.

**Table 3:** Health state utilities and disutilities

Health state	Utility			Disutility of symptoms/impairment (difference from ASMD without impairment)^a^		
	Mean	(SD)	95% CI	Mean	(SD)	95% CI
Adult ASMD health states						
A1: No symptoms or impairment	0.918	(0.184)	0.892 to 0.943	–	–	–
A2: Mild-to-moderate pulmonary dysfunction	0.847	(0.215)	0.817 to 0.876	− 0.071	(0.110)	− 0.086 to − 0.056
A3: Moderate spleen/liver enlargement	0.837	(0.219)	0.806 to 0.867	− 0.081	(0.116)	− 0.097 to − 0.065
A4: Mild-to-moderate ASMD	0.770	(0.246)	0.736 to 0.804	− 0.148	(0.166)	− 0.171 to − 0.125
A5: Mild-to-moderate pulmonary dysfunction with severe spleen/liver enlargement	0.532	(0.423)	0.474 to 0.591	− 0.385	(0.381)	− 0.438 to − 0.332
A6: Severe pulmonary dysfunction with moderate spleen/liver enlargement	0.527	(0.402)	0.472 to 0.583	− 0.390	(0.359)	− 0.440 to − 0.340
A7: Severe ASMD	0.333	(0.468)	0.268 to 0.398	− 0.584	(0.440)	− 0.645 to − 0.523
Child ASMD health states						
C1: No symptoms or impairment	0.939	(0.104)	0.924 to 0.953	–	–	–
C2: Mild-to-moderate pulmonary dysfunction	0.882	(0.146)	0.862 to 0.903	− 0.056	(0.092)	− 0.069 to − 0.044
C3: Moderate spleen/liver enlargement	0.867	(0.191)	0.840 to 0.893	− 0.072	(0.120)	− 0.088 to − 0.055
C4: Mild-to-moderate ASMD	0.809	(0.241)	0.775 to 0.842	− 0.130	(0.182)	− 0.155 to − 0.104
C5: Mild-to-moderate pulmonary dysfunction with severe spleen/liver enlargement	0.594	(0.374)	0.542 to 0.646	− 0.344	(0.335)	− 0.391 to − 0.298
C6: Severe pulmonary dysfunction with moderate spleen/liver enlargement	0.654	(0.346)	0.606 to 0.702	− 0.285	(0.301)	− 0.326 to − 0.243
C7: Severe ASMD	0.450	(0.431)	0.390 to 0.510	− 0.489	(0.396)	− 0.544 to − 0.434

*ASMD* acid sphingomyelinase deficiency, *CI* confidence interval, *SD* standard deviation

^a^Disutility of each symptom/impairment is computed by subtracting health state 1 (ASMD without impairment) from each of the other health states (ASMD with impairment)

To estimate disutility associated with various severity levels of ASMD symptoms, utility differences were calculated for all health states with symptoms (i.e., A2 to A7 and C2 to C7) compared to ASMD without symptoms or impairment (A1 and C1). These disutilities are presented in Table 3. Utility differences for the adult health states ranged from − 0.07 for mild-to-moderate breathing impairment (A2) to − 0.58 for severe ASMD (A7). Disutilities for the child health states ranged from − 0.06 for mild-to-moderate breathing impairment (C2) to − 0.49 for severe ASMD (C7). Paired *t* tests found that utilities of all health states with symptoms were significantly lower than the utility of ASMD without impairment (all *P* < 0.0001).

Willingness to trade time varied by health state severity. Most participants were not willing to trade time to avoid the adult (69.8%) and pediatric (68.3%) health states describing ASMD without symptoms or impairment (A1, C1). However, more than half of the participants were willing to trade time to avoid all other health states, with over 90% willing to trade time to avoid the severe ASMD health states (A7, C7).

Most participants rated all adult and child health states as preferable to dead, resulting in positive utilities. Severe ASMD health states A7 and C7 had the highest rates of negative utilities, with 13.4% and 8.9% perceiving these health states to be worse than dead.

The adult and child health states each received a total of 1414 valuations (i.e., seven health states valued by 202 respondents). For the adult health states, there were a total of 63 negative valuations (4.5%) and 27 (1.9%) valuations resulting in a utility of 1. For the child health states, there were a total of 42 negative valuations (3.0%) and 26 (1.8%) valuations resulting in a utility of 1.

### Subgroup analyses

There were no significant between-group differences in utility by gender for either the adult or child health states. In addition, there were no significant age differences for utilities of the adult health states (i.e., older vs. younger subgroups, categorized based on median split). However, there were differences by age for the child health states. For all child health states, the older subgroup (*n* = 99) had greater mean utilities than the younger subgroup (*n* = 103), and this between-group difference was statistically significant for four health states (C2, C3, C4, C7), with the greatest difference for the severe ASMD health state (0.120, *P* = 0.047).

These age differences for the child health states may be related to whether the respondents had children. Among the older subgroup, 44 of 99 respondents (44.4%) reported having children. In contrast, only seven of the 103 respondents (6.8%) in the younger subgroup reported having children. To examine the potential association between utilities and parenting status, *t* tests were conducted to compare utilities between the subgroups with (*n* = 51) and without (*n* = 151) children (Table 4). There were no significant between-group differences for the adult health states. However, the groups diverged in valuations of the child health states. The subgroup with children gave higher utilities for every child health state. The between-group difference was statistically significant for five of the seven health states (C3 to C7), and this difference increased with the more severe health states. The severe ASMD health state had the greatest utility difference (between-group difference = 0.21, *P* = 0.002).

**Table 4:** Comparison of health state utilities valued by participants with and without children^a^

Health state utilities	With children (*n* = 51) Mean (SD)	Without children (*n* = 151) Mean (SD)	Difference	*t* test	
			Mean (SD)	*t* statistic	*P* value
Adult ASMD health states					
A1: No symptoms or impairment	0.91 (0.13)	0.92 (0.20)	− 0.01 (0.18)	− 0.4	0.707
A2: Mld-to-moderate pulmonary dysfunction	0.84 (0.19)	0.85 (0.22)	− 0.01 (0.22)	− 0.4	0.691
A3: Moderate spleen/liver enlargement	0.85 (0.19)	0.83 (0.23)	0.02 (0.22)	0.4	0.671
A4: Mild-to-moderate ASMD	0.80 (0.20)	0.76 (0.26)	0.03 (0.25)	0.8	0.399
A5: Mild-to-moderate pulmonary dysfunction with severe spleen/liver enlargement	0.58 (0.39)	0.52 (0.43)	0.06 (0.42)	0.9	0.376
A6: Severe pulmonary dysfunction with moderate spleen/liver enlargement	0.53 (0.44)	0.53 (0.39)	0.01 (0.40)	0.1	0.897
A7: Severe ASMD	0.39 (0.50)	0.32 (0.46)	0.07 (0.47)	0.9	0.352
Child health states					
C1: No symptoms or impairment	0.95 (0.06)	0.93 (0.12)	0.01 (0.10)	1.2	0.242
C2: Mild-to-moderate pulmonary dysfunction	0.91 (0.12)	0.87 (0.15)	0.03 (0.15)	1.6	0.118
C3: Moderate spleen/liver enlargement	0.90 (0.12)	0.85 (0.21)	0.05 (0.19)	2.0	0.048
C4: Mild-to-moderate ASMD	0.87 (0.15)	0.79 (0.26)	0.09 (0.24)	2.9	0.005
C5: Mild-to-moderate pulmonary dysfunction with severe spleen/liver enlargement	0.70 (0.33)	0.56 (0.38)	0.15 (0.37)	2.4	0.016
C6: Severe pulmonary dysfunction with moderate spleen/liver enlargement	0.75 (0.30)	0.62 (0.36)	0.12 (0.34)	2.2	0.028
C7: Severe ASMD	0.61 (0.40)	0.40 (0.43)	0.21 (0.42)	3.1	0.002

*ASMD* acid sphingomyelinase deficiency, *SD* standard deviation

^a^Participants with children answered “Yes” to “Do you have any children?” Participants without children answered “No” to this question

### Child considered when valuing the pediatric health states

For the pediatric health states, respondents were asked to think about the health states in the context of “an 8-year-old child with a rare genetic disorder.” After completing the TTO utility elicitation, respondents were asked whether they were thinking of a specific child. Responses varied widely (Table 5). Approximately half of the participants who had children (51.0%) reported thinking about their own child when valuing the pediatric health states. In contrast, most (64.2%) of the participants without children thought about a hypothetical or generic child rather than a specific child.

**Table 5:** Child considered when rating pediatric health states (*n* = 202)

“When you were rating the health states of an 8-year-old child, were you thinking of a specific child?”^a^, *n* (%)	Frequency, *n* (%)		
	Participants with children (*n* = 51)	Participants without children (*n* = 151)	Total sample (*n* = 202)
Own child	26 (51.0%)	–	26 (12.9%)
Myself as a child	6 (11.8%)	17 (11.3%)	23 (11.4%)
Generic/random/non-specific/hypothetical child	19 (37.3%)	97 (64.2%)	116 (57.4%)
A child they know who has a disease/disorder that is not ASMD	–	6 (4.0%)	6 (3.0%)
Own hypothetical child	–	8 (5.3%)	8 (4.0%)
Niece/nephew	–	13 (8.6%)	13 (6.4%)
Cousin	–	5 (3.3%)	5 (2.5%)
Grandchild	2 (3.9%)	–	2 (1.0%)
Child(ren) who I teach/nanny	1 (2.0%)	2 (1.3%)	3 (1.5%)
Child(ren) of friends	1 (2.0%)	1 (0.7%)	2 (1.0%)
Other child^b^	2 (3.9%)	6 (4.0%)	8 (4.0%)
Missing	–	1 (0.7%)	1 (0.5%)

*ASMD* acid sphingomyelinase deficiency

^a^This was the question as phrased to participants. Some participants provided more than one response

^b^Other child: “A girl- to distance myself from them as a man” (*n* = 1), “A Palestinian child living in a rough area” (*n* = 1), “Children in school” (*n* = 1), “Ethnic child” (*n* = 1), “Godson who is 8” (*n* = 1), “My partner’s son is 8 years old so I picked him” (*n* = 1), “Children at school that had larger tummies when I was at school and how it impacted them” (*n* = 1), and “Younger brother” (*n* = 1)

## Discussion

Utilities followed the expected pattern with greater severity of ASMD associated with lower mean utility for both the adult and pediatric health states. Consistent with the broad range of disease severity in these health states, utilities varied widely. Scores ranged from 0.92/0.94 (i.e., adult/child) for ASMD without symptoms or impairment to 0.33/0.45 for ASMD with severe impairment. While the higher utilities are in the range typically observed for relatively healthy individuals, the lower utilities are in a range similar to other diseases with substantial symptom severity and impairment, such as progressive lymphoma [60] and short bowel syndrome with daily intravenous nutrition supplementation [61].

This study provides seven health state utilities corresponding to various severities of ASMD in children and another seven utilities for severities of ASMD in adults. These 14 values may be used to represent the health status of patients with ASMD in cost-utility modeling. For example, a utility of 0.450 may be used in a model to represent a child with severe ASMD prior to receiving treatment. If a clinical trial demonstrates that pediatric patients with severe ASMD typically improve to mild-to-moderate ASMD with treatment, the utility of 0.809 may be used to represent the hypothetical child at endpoint. Utilities for both children and adults are presented in Table 3.

Because of the study design, the current study offers a unique opportunity to compare utility elicitation for a parallel set of adult and child health states, valued by the same respondents. The difference between adult and child health states varied across participants. Some respondents had higher utilities for adult health states, while others had higher utilities for child health states. However, for all seven health state pairs, it was more common for respondents to trade more time from their own lives than from the life of a child, resulting in lower utilities for the adult health state (see Fig. 1 for an illustration of this trend). For example, for health states A6 and C6, 59.9% of the sample had a higher utility for the child health state (C6), 25.7% had a higher utility for the adult health state (A6), and 14.4% had the same utility for both.

This utility difference between adult and child health states is consistent with results from previous studies in which EQ-5D-3L and EQ-5D-Y health states were valued by adult general population respondents [62, 63]. In these previous studies, utilities tended to be lower when respondents were asked to imagine themselves as adults living in the EQ-5D health states, compared with considering a child living in the same health states. The current study is the first to show that this trend occurs with utilities of disease-specific adult and child health state vignettes valued by the same respondents. In addition, the current results suggest that the difference between utilities for adult and child health states may tend to increase as the disease becomes more severe and utility values decrease.

Follow-up analyses highlight subgroup differences in evaluation of child health states. Previous studies have found that being a parent or caregiver of a child has an impact on TTO valuations, tending to reduce the amount of time people are willing to trade, which leads to higher utilities [64, 65]. Results for most of the adult health states in the current study followed this pattern, but with relatively small and non-significant differences between subgroups with and without children (Table 4). For the child health states, this between-group difference was more consistent, greater in magnitude, and statistically significant for the five most severe health states.

To explore reasons for this between-group difference, interviewers asked respondents who they were imagining when valuing the child health states. While the majority (64.2%) of participants without children were not thinking of a specific child, over half of the participants with children were thinking of their own child. This suggests that having a child has an impact on respondents’ approach to valuing hypothetical pediatric health states. In a recently published study with EQ-5D health states, Powell et al. observed a similar pattern and suggested that rates of parenting status should be considered when recruiting a sample for valuation of child health [63]. Current results offer further support for this conclusion and show that this pattern of findings occurs not only with EQ-5D health states but also condition-specific vignettes. When recruiting a sample to value child health in TTO tasks, researchers can try not to over- or under-represent parents in the sample relative to the general population. Unfortunately, this is not a straightforward task because it is challenging to identify clear statistics on rates of parenthood in the general population. Rates of parenthood vary by age, sex, and country, and statistics may not be available for all groups. Still, some consideration of available statistics may be useful for identifying broad parenting status targets [66].

Results of the current study should be interpreted in the context of several limitations. As described previously [37], vignette-based methods have inherent limitations because the resulting utilities represent preferences for health state vignettes, rather than the experiences of patients living in the health states. To minimize these limitations, health states were developed carefully based on a range of perspectives, including input from multiple clinicians, patients with ASMD, and caregivers of pediatric patients with ASMD. All agreed that the health states were an accurate representation of patients’ typical experiences with this disease. Furthermore, a patient-based approach such as the EQ-5D would not have been feasible for a rare disease where it is difficult or impossible to recruit a sufficient number of patients within each disease severity health state. Still, the extent to which current vignette-based utilities may be consistent with utilities derived directly from patients is not known.

There are also limitations associated with the study sample. Due to the COVID-19 pandemic, several decisions were made that could affect generalizability of this sample. Because of the complexity and number of health states (14 health states in total, including seven adult and seven child), the study team believed it was essential to conduct interviews in person rather than via videoconferencing. The initial plan was to conduct interviews in multiple locations in the UK in December 2021. However, because the omicron variant emerged at that time, interviews were delayed until March 2022. In addition, interviews were conducted in only one location (London) to minimize increased COVID-19 risks associated with travel. Therefore, while the sample was selected to be representative for age, gender, ethnicity and employment, the sample was geographically restricted to a single location.

Furthermore, to minimize risk of COVID-19 transmission, all interviewers and participants were required to have been vaccinated and wear a mask during the interviews. This could have resulted in sample selection bias because values of people from the general population who preferred not to be vaccinated or wear a mask are not represented in the current study. It is possible that people who prefer not to be vaccinated may tend to respond differently in a TTO task than people who choose to be vaccinated.

Despite limitations, the utilities estimated in this study would be useful in economic models evaluating the cost-effectiveness of treatments for adult and pediatric patients with ASMD. Results also have implications for evaluation of pediatric health states. The utility differences between adult and child health states found in this study support findings from previous research suggesting that utilities derived for adult health states cannot necessarily be used in models evaluating cost-effectiveness of treatments for children. This is particularly important for more severe disease states where there were significant utility differences between health states representing adults and children with the same medical condition.

## Supplementary Information

Below is the link to the electronic supplementary material.

Supplementary file1 (DOCX 29 KB)

## Data Availability

Data will be made available upon reasonable request.
