# Supplementary material for: Assessment of health state utilities associated with adult and pediatric acid sphingomyelinase deficiency (ASMD)
Source: Eur J Health Econ. 2024 Feb 27;25(8):1437–48. doi: 10.1007/s10198-023-01667-7 (PMC11442559; doi:10.1007/s10198-023-01667-7)
Supplement: Supplementary file 1 — Supplementary file1 (DOCX 29 KB) [file 10198_2023_1667_MOESM1_ESM.docx]

# SUPPLEMENTARY MATERIAL: HEALTH STATE VIGNETTES

# Adult Health States

## Background Information: Adult

**Disease Description**

- You have a **rare** **genetic disorder** in which cells have difficulty breaking down a type of fat that exists in everyone’s body.
- Over time, this fat accumulates in organs, including the lungs, spleen, and liver.
- The **lungs** absorb oxygen from the air that is breathed and transfer this oxygen to the bloodstream so that it can get to every part of the body.
- The **spleen** is an organ in the abdomen (tummy). It is a part of the blood system that stores some types of blood cells. It is also involved in the immunological system that protects you from disease.
- The **liver** is an organ in the abdomen (tummy). The liver has a variety of functions related to your metabolism. For example, it filters the blood and breaks down harmful substances. The liver also helps control blood sugar, and it makes important proteins such as the ones that help your blood clot.
- As **this fat accumulates** in the lungs, spleen, and liver, it can cause a **variety of symptoms**.

**Seven Health States**

- You will review seven health states, each describing what it is like for an adult to live with this genetic disorder.
- The health states vary in severity.

**Health State A1**

**Breathing: No Impairment**

- You do not have trouble breathing.

**Spleen and Liver: Normal**

- Your spleen and liver are not noticeably larger than normal.

**Quality of Life**

***Activities***

- You can perform your **daily** **activities** **without difficulty**.
- Your health condition does not have any impact on your ability to **walk around**.
- Your doctor tells you to avoid activities that could damage your spleen, such as contact sports or mountain biking.
- Your medical condition does not limit your ability to work.

***Infections and Hospitalisation***

- You do not get infections or require hospitalisation more often than other people.

***Appearance***

- Your health condition does not have an impact on your appearance.

***Emotional Impact***

- You are sometimes concerned about the future of your health.

**Health State A2**

**Breathing: Mild to Moderate Impairment**

- You feel **short of breath** more easily than most people.
- Taking a deep breath feels **a little** **uncomfortable**, like a tightness or aching in the chest. You notice this when you feel short of breath.
- Sometimes it is **difficult** to talk while walking because of shortness of breath.

**Spleen and Liver: Normal**

- Your spleen and liver are not noticeably larger than normal.

**Quality of Life**

***Activities***

- Your health condition interferes with **some** **activities** when you feel short of breath.
- You **may need to stop and rest** after climbing stairs or walking uphill.
- Your doctor tells you to avoid activities that could damage your spleen, such as contact sports or mountain biking.
- Your symptoms limit your ability to work at a job involving physical labour.

***Infections and Hospitalisation***

- You do not get infections or require hospitalisation more often than other people.

***Appearance***

- Your health condition does not have an impact on your appearance.

***Emotional Impact***

- You are sometimes concerned about the future of your health.

**Health State A3**

**Breathing: No Impairment**

- You do not have trouble breathing.

**Spleen and Liver: Moderately Enlarged**

- Your spleen and liver are **moderately** **enlarged**.
- You **bruise easily** and get **occasional nosebleeds**.
- You **sometimes** have **moderate abdominal discomfort.** For example, you notice this when bending over to put on shoes. You have **rare** episodes of more severe pain.
- You feel **tired** more often than most people.
- When eating, you feel full before eating a normal amount of food. This is **sometimes** **uncomfortable**.

**Quality of Life**

***Activities***

- Your health condition interferes with **some** **activities** when you feel uncomfortable or tired.
- Your health condition usually does not have any impact on your ability to **walk around**.
- Your doctor tells you to avoid activities that could damage your spleen, such as contact sports or mountain biking.
- Sometimes, you may need to **take time off work** because of symptoms.

***Infections and Hospitalisation***

- You do not get infections or require hospitalisation more often than other people.

***Appearance***

- You have a **larger tummy**. This is **noticeable** to other people.

***Emotional Impact***

- You are sometimes concerned about the future of your health.

**Health State A4**

**Breathing: Mild to Moderate Impairment**

- You feel **short of breath** more easily than most people.
- Taking a deep breath feels **a little** **uncomfortable**, like a tightness or aching in the chest. You notice this when you feel short of breath.
- Sometimes it is **difficult** to talk while walking because of shortness of breath.

**Spleen and Liver: Moderately Enlarged**

- Your spleen and liver are **moderately** **enlarged**.
- You **bruise easily** and get **occasional** **nosebleeds**.
- You **sometimes** have **moderate abdominal discomfort**. For example, you notice this when bending over to put on shoes. You have **rare** episodes of more severe pain.
- You feel **tired** more often than most people.
- When eating, you feel full before eating a normal amount of food. This is **sometimes** **uncomfortable**.

**Quality of Life**

***Activities***

- Your health condition interferes with **some** **activities** when you feel short of breath, uncomfortable, or tired.
- You **may need to stop and rest** after climbing stairs or walking uphill.
- Your doctor tells you to avoid activities that could damage your spleen, such as contact sports or mountain biking.
- Sometimes, you need to **take time off work** because of symptoms.
- Your symptoms limit your ability to work at a job involving physical labour.

***Infections and Hospitalisation***

- You do not get infections or require hospitalisation more often than other people.

***Appearance***

- You have a **larger tummy**. This is **noticeable** to other people.

***Emotional Impact***

- You are sometimes concerned about the future of your health.

**Health State A5**

**Breathing: Mild to Moderate Impairment**

- You feel **short of breath** more easily than most people.
- Taking a deep breath feels **a little** **uncomfortable**, like a tightness or aching in the chest. You notice this when you feel short of breath.
- Sometimes it is **difficult** to talk while walking because of shortness of breath.

**Spleen and Liver: Very Enlarged**

- Your spleen and liver are **very enlarged**.
- You **bruise easily**, and these bruises are often large and painful. You get **frequent nosebleeds** that are often difficult to stop.
- You **often** have **moderate abdominal discomfort.** For example, you notice this when bending over to put on shoes. You have **occasional** episodes of more severe pain.
- You feel **tired most days**, and this affects many of your daily activities.
- When eating, you feel full before eating a normal amount of food. This is **often uncomfortable**, and you sometimes feel nausea.

**Quality of Life**

***Activities***

- Your health condition interferes with **many activities** because of shortness of breath and feeling uncomfortable or tired.
- You **may** **need to stop and rest** after climbing stairs or walking uphill.
- You **cannot exercise or play sports**.
- You need **often need to take time off work** because of symptoms and infections.
- You cannot work at a job involving physical labour.

***Infections and Hospitalisation***

- You are **more** **susceptible to infections**. This typically happens **once or twice per year**, and it is treated with antibiotics.
- About **once per year**, you are **admitted to** **hospital** for severe episodes of **abdominal pain**.

***Appearance***

- You have a **very large tummy.** This is **obviously** **noticeable** to other people.

***Emotional Impact***

- You are anxious about the future of your health.

**Health State A6**

**Breathing: Severe Impairment**

- You feel **short of breath** with most activities.
- Taking a deep breath feels **very uncomfortable**, like a tightness or aching in the chest. You notice this when you feel short of breath. You **avoid activities** that make you feel this way.
- You **cannot comfortably talk while walking** because of shortness of breath.

**Spleen and Liver: Moderately Enlarged**

- Your spleen and liver are **moderately** **enlarged**.
- You **bruise easily** and get **occasional** **nosebleeds**.
- You **sometimes** have **moderate abdominal discomfort.** For example, you notice this when bending over to put on shoes. You have **rare** episodes of more severe pain.
- You feel **tired** more often than most people.
- When eating, you feel full before eating a normal amount of food. This is **sometimes** **uncomfortable**.

**Quality of Life**

***Activities***

- Your health condition interferes with **most activities** because of shortness of breath and feeling uncomfortable or tired.
- You **cannot climb a flight of stairs without resting** on the way up. When walking on flat ground, you **often need to stop and rest**.
- You **cannot exercise or play sports**.
- You **often need to take time off work** because of symptoms and infections.
- You cannot work at a job involving physical labour.

***Infections and Hospitalisation***

- You are **more susceptible to infections**. This typically happens **once or twice per year**, and it is treated with antibiotics.
- You **do not** require hospitalisation more often than other people.

***Appearance***

- You have a **larger tummy**. This is **noticeable** to other people.

***Emotional Impact***

- You are anxious about the future of your health.

**Health State A7**

**Breathing: Severe Impairment**

- You feel **short of breath** with most activities.
- Taking a deep breath feels **very uncomfortable**, like a tightness or aching in the chest. You notice this when you feel short of breath. You **avoid activities** that make you feel this way.
- You **cannot comfortably talk while walking** because of shortness of breath.

**Spleen and Liver: Very Enlarged**

- Your spleen and liver are **very enlarged**.
- You **bruise easily**, and these bruises are often large and painful. You get **frequent** **nosebleeds** that are often difficult to stop.
- You **often** have **moderate abdominal discomfort**. For example, you notice this when bending over to put on shoes. You have **occasional** episodes of more severe pain.
- You feel **tired most days**, and this affects many of your daily activities.
- When eating, you feel full before eating a normal amount of food. This **is often uncomfortable**, and you sometimes feel nausea.

**Quality of Life**

***Activities***

- Your health condition interferes with **most activities** because of shortness of breath and feeling uncomfortable or tired.
- You **cannot climb a flight of stairs without resting** on the way up. When walking on flat ground, you **often need to stop and rest**.
- You **cannot exercise or play sports**.
- You **often need to take time off work** because of symptoms and infections.
- You cannot work at a job involving physical labour.

***Infections and Hospitalisation***

- You are **more susceptible to infections**. This typically happens **once or twice per year**, and it is treated with antibiotics.
- About **once per year**, you are **admitted to** **hospital** for severe episodes of **abdominal pain**.

***Appearance***

- You have a **very large tummy.** This is **obviously noticeable** to other people.

***Emotional Impact***

- You are anxious about the future of your health.

# Child Health States

## Background Information: Child

**Disease Description**

- This child has a **rare genetic disorder** in which cells have difficulty breaking down a type of fat that exists in everyone’s body.
- Over time, this fat accumulates in organs, including the lungs, spleen, and liver.
- The **lungs** absorb oxygen from the air that is breathed and transfer this oxygen to the bloodstream so that it can get to every part of the body.
- The **spleen** is an organ in the abdomen (tummy). It is a part of the blood system that stores some types of blood cells. It is also involved in the immunological system that protects you from disease.
- The **liver** is an organ in the abdomen (tummy). The liver has a variety of functions related to your metabolism. For example, it filters the blood and breaks down harmful substances. The liver also helps control blood sugar, and it makes important proteins such as the ones that help your blood clot.
- As **this fat accumulates** in the lungs, spleen, and liver, it can cause a **variety of symptoms**.

**Seven Health States**

- You will review seven health states, each describing what it is like for a child to live with this genetic disorder.
- The health states vary in severity.

**Health State C1**

**Breathing: No Impairment**

- The child does not have trouble breathing.

**Spleen and Liver: Normal**

- The child’s spleen and liver are not noticeably larger than normal.

**Quality of Life**

***Activities***

- The child is able to perform their **daily** **activities** **without difficulty**.
- The child’s health condition does not have any impact on their ability to **walk around**.
- The doctor tells the child to avoid athletic activities that could damage the spleen, such as playing contact sports with friends.
- The child’s health condition does not limit their ability to go to school.

***Infections and Hospitalisation***

- The child does not get infections or require hospitalisation more often than other children.

***Appearance***

- The child is **a little smaller** than their peers.

***Emotional Impact***

- The child is sometimes concerned about the future of their health.

**Health State C2**

**Breathing: Mild to Moderate Impairment**

- The child feels **short of breath** more easily than most children.
- Taking a deep breath feels **a little uncomfortable**, like a tightness or aching in the chest. The child notices this when they feel short of breath.
- Sometimes it is **difficult** to talk while walking because of shortness of breath.

**Spleen and Liver: Normal**

- The child’s spleen and liver are not noticeably larger than normal.

**Quality of Life**

***Activities***

- The child’s health condition interferes with **some** **activities** when they feel short of breath.
- The child **may** **need to stop and rest** after climbing stairs or walking uphill.
- It is difficult for the child to play games that involve **running**.
- The doctor tells the child to avoid athletic activities that could damage the spleen, such as playing contact sports with friends.
- Sometimes, the child **stays home from school** because of symptoms.

***Infections and Hospitalisation***

- The child does not get infections or require hospitalisation more often than other children.

***Appearance***

- The child is **a little smaller** than their peers.

***Emotional Impact***

- The child is sometimes concerned about the future of their health.

**Health State C3**

**Breathing: No Impairment**

- The child does not have trouble breathing.

**Spleen and Liver: Moderately Enlarged**

- The child’s spleen and liver are **moderately enlarged**.
- The child **bruises easily** and gets **occasional** **nosebleeds**.
- The child **sometimes** has **moderate abdominal discomfort.** For example, the child notices this when bending over to put on shoes. The child has **rare** episodes of more severe pain.
- The child feels **tired** more often than most children.
- When eating, the child feels full before eating a normal amount of food. This is **sometimes** **uncomfortable**.

**Quality of Life**

***Activities***

- The child’s health condition interferes with **some activities** when they feel uncomfortable or tired.
- The child’s health condition does not have any impact on their ability to **walk around**.
- The doctor tells the child to avoid athletic activities that could damage the spleen, such as playing contact sports with friends.
- Sometimes, the child **stays home from school** because of symptoms.

***Infections and Hospitalisation***

- The child does not get infections or require hospitalisation more often than other children.

***Appearance***

- The child has a **larger tummy.** This is **noticeable** to other people.
- The child is **a little smaller** than their peers.

***Emotional Impact***

- The child is sometimes concerned about the future of their health.

**Health State C4**

**Breathing: Mild to Moderate Impairment**

- The child feels **short of breath** more easily than most children.
- Taking a deep breath feels **a little uncomfortable**, like a tightness or aching in the chest. The child notices this when they feel short of breath.
- Sometimes it is **difficult** to talk while walking because of shortness of breath.

**Spleen and Liver: Moderately Enlarged**

- The child’s spleen and liver are **moderately enlarged**.
- The child **bruises easily** and gets **occasional** **nosebleeds**.
- The child **sometimes** has **moderate abdominal discomfort.** For example, the child notices this when bending over to put on shoes. The child has **rare** episodes of more severe pain.
- The child feels **tired** more often than most children.
- When eating, the child feels full before eating a normal amount of food. This is **sometimes** **uncomfortable**.

**Quality of Life**

***Activities***

- The child’s health condition interferes with **some** **activities** when they feel short of breath, uncomfortable, or tired.
- The child **may** **need to stop and rest** after climbing stairs or walking uphill.
- It is difficult for the child to play games that involve **running**.
- The doctor tells the child to avoid athletic activities that could damage the spleen, such as playing contact sports with friends.
- Sometimes, the child **stays home from school** because of symptoms.

***Infections and Hospitalisation***

- The child does not get infections or require hospitalisation more often than other children.

***Appearance***

- The child has a **larger tummy**. This is **noticeable** to other people.
- This child is **a little smaller** than their peers.

***Emotional Impact***

- The child is sometimes concerned about the future of their health.

**Health State C5**

**Breathing: Mild to Moderate Impairment**

- The child feels **short of breath** more easily than most children.
- Taking a deep breath feels **a little uncomfortable**, like a tightness or aching in the chest. The child notices this when they feel short of breath.
- Sometimes it is **difficult** to talk while walking because of shortness of breath.

**Spleen and Liver: Very Enlarged**

- The child’s spleen and liver are **very enlarged**.
- The child **bruises easily**, and these bruises are often large and painful. The child gets **frequent nosebleeds** that are often difficult to stop.
- The child **often** has **moderate abdominal discomfort.** For example, the child notices this when bending over to put on shoes. The child has **occasional** episodes of more severe pain.
- The child feels **tired** **most days**, and this affects many of their daily activities.
- When eating, the child feels full before eating a normal amount of food. This is **often uncomfortable**, and the child sometimes feels nausea.
- Occasionally, the child avoids eating.

**Quality of Life**

***Activities***

- The child’s health condition interferes with **many activities** because of shortness of breath and feeling uncomfortable or tired.
- The child **may** **need to stop and rest** after climbing stairs or walking uphill.
- It is difficult for the child to play games that involve **running**.
- The doctor tells the child to avoid athletic activities that could damage the spleen, such as playing contact sports with friends.
- The child **often misses school** because of symptoms and infections.

***Infections and Hospitalisation***

- The child is **more susceptible to nosebleeds and infections**.
- The child stays **in hospital once or twice each year** because of a nosebleed or an infection. This usually lasts less than a week. The infection is treated with antibiotics.

***Appearance***

- The child has a **very large tummy.** This is **obviously noticeable** to other people.
- The child is **much smaller** than their peers and has **less muscle strength** than other children.

***Emotional Impact***

- The child is anxious about the future of their health.

**Health State C6**

**Breathing: Severe Impairment**

- The child feels **short of breath** with most activities.
- Taking a deep breath feels **very uncomfortable**, like a tightness or aching in the chest. The child notices this when they feel short of breath. The child **avoids activities** that make them feel this way.
- The child **cannot comfortably talk while walking** because of shortness of breath.

**Spleen and Liver: Moderately Enlarged**

- The child’s spleen and liver are **moderately enlarged**.
- The child **bruises easily** and gets **occasional** **nosebleeds**.
- The child **sometimes** has **moderate abdominal discomfort.** For example, the child notices this when bending over to put on shoes. The child has **rare** episodes of more severe pain.
- The child feels **tired** more often than most children.
- When eating, the child feels full before eating a normal amount of food. This is **sometimes** **uncomfortable**.

**Quality of Life**

***Activities***

- The child’s health condition interferes with **most activities** because of shortness of breath and feeling uncomfortable or tired.
- The child **cannot climb a flight of stairs without resting** on the way up. When walking on flat ground, the child **often needs to stop and rest**.
- The child **cannot run**.
- The doctor tells the child to avoid athletic activities that could damage the spleen, such as playing contact sports with friends.
- The child **often misses school** because of symptoms and infections.

***Infections and Hospitalisation***

- The child is **more susceptible to infections.**
- The child **stays in hospital once or twice each year** because of an **infection**, including chest infections with symptoms of cough, fever, and producing phlegm. This is treated with antibiotics and usually lasts less than a week.

***Appearance***

- The child has a **larger tummy**. This is **noticeable** to other people.
- The child is **much smaller** than their peers and has **less muscle strength** than other children.

***Emotional Impact***

- The child is anxious about the future of their health.

**Health State C7**

**Breathing: Severe Impairment**

- The child feels **short of breath** with most activities.
- Taking a deep breath feels **very uncomfortable**, like a tightness or aching in the chest. The child notices this when they feel short of breath. The child **avoids activities** that make them feel this way.
- The child **cannot comfortably talk while walking** because of shortness of breath.

**Spleen and Liver: Very Enlarged**

- The child’s spleen and liver are **very enlarged**.
- The child **bruises easily**, and these bruises are often large and painful. The child gets **frequent nosebleeds** that are often difficult to stop.
- The child **often** has **moderate abdominal discomfort.** For example, the child notices this when bending over to put on shoes. The child has **occasional** episodes of more severe pain.
- The child feels **tired** **most days**, and this affects many of their daily activities.
- When eating, the child feels full before eating a normal amount of food. This is **often uncomfortable**, and the child sometimes feels nausea.
- Occasionally, the child avoids eating.

**Quality of Life**

***Activities***

- The child’s health condition interferes with **most** **activities** because of shortness of breath and feeling uncomfortable or tired.
- The child **cannot climb a flight of stairs without resting** on the way up. When walking on flat ground, the child **often needs to stop and rest**.
- The child **cannot run**.
- The doctor tells the child to avoid athletic activities that could damage the spleen, such as playing contact sports with friends.
- The child **often misses school** because of symptoms and infections.

***Infections and Hospitalisation***

- The child is **more susceptible to nosebleeds** and **infections** including chest infections with symptoms of cough, fever, and producing phlegm.
- The child stays in **hospital once or twice each year** because of a nosebleed or an infection. This usually lasts less than a week. The infection is treated with antibiotics.

***Appearance***

- The child has a **very large tummy.** This is **obviously noticeable** to other people.
- The child is **much smaller** than their peers and has **less muscle strength** than other children.

***Emotional Impact***

- The child is anxious about the future of their health.
